# Supplementary material for: Chromothripsis during telomere crisis is independent of NHEJ, and consistent with a replicative origin
Source: Genome Res. 2019 May;29(5):737–49. doi: 10.1101/gr.240705.118 (PMC6499312; doi:10.1101/gr.240705.118)
Supplement: Supplemental Material [file supp_gr.240705.118_Supplemental_file_1.zip › contigs/annotated_contigs/DB53/contig.2.DB53_length_578_mean_cov_4.05190311419.docx]

**DB53_length_578_mean_cov_4.05190311419**

```
ATGGTATAAATACAAGCATTATGCTGAACTCTTTGCCAGAGGAAATAGAATGCAGTCTTTTGTTCTCAGGTCCAAGATTGGGAGAGAGA
 >chr8:132144639-132144849 - E=1e-114
TTGGGTCAGGTAACATTCAGGTTCCCTTCAAACTTTTAGACCCTAACTACACTTTTCTTTCCCTCTACCCTATTTATTGCCAGCCAAAG

GCCTCATGGAAGAGTCCTGTAGCCCAG|CAAAG|CTGCATTTATCACACACATTGATGTTTCTTACGTGCTTAATAACCTTTTTAGGAA
                            >chr8:132143892-132144265 + E=7e-210
TGGGATTTTAGGGTCAAAATACATTGTGGGAGTGGATGCATGTATATTATCATACTGCCTTCCAAAAGGATGTATCTCACATTCTCATC

AATATTTTGTAAGAGCGGCCATCCCCACCCCTCCATTGCTACAATTATTTTTAAAAATTATTTTAAATTTTATTTTTAAAAAATTAGTT

AAAATAAACCAAAAGTATGTATCACCTCAAATAGGAACTTATCCTTAATTCAATTAGAGCCATGTTAACATTGAGTTATGTGGGGGATA

GGGAAACAGTTTGGTTTTTCTGTCACTTTCACCCAGTATCAATGGG
```
